# Management of vasomotor symptoms in cancer patients

**DOI:** 10.1093/oncolo/oyaf002

**Published:** 2025-03-04

**Authors:** Ling Zhu, Tammy T Hshieh, Tara K Iyer, Alicia K Morgans, Ole-Petter R Hamnvik

**Affiliations:** Department of Endocrinology, Singapore General Hospital, Singapore 169856; Division of Endocrinology, Diabetes and Hypertension, Department of Medicine, Brigham and Women’s Hospital, Boston, MA 02115, United States; Division of Aging, Department of Medicine, Brigham and Women’s Hospital, Boston, MA 02115, United States; Menopause and Midlife Clinic, Division of Women’s Health, Department of Medicine, Brigham and Women’s Hospital, Boston, MA 02215, United States; Lank Center for Genitourinary Oncology, Dana-Farber Cancer Institute, Boston, MA 02215, United States; Division of Endocrinology, Diabetes and Hypertension, Department of Medicine, Brigham and Women’s Hospital, Boston, MA 02115, United States

**Keywords:** hot flashes, menopause, breast cancer, prostate cancer, cancer survivors

## Abstract

Many cancer treatments can lead to reduced levels of sex hormones, which in turn may cause vasomotor symptoms (VMS) such as hot flashes. These symptoms are associated with impaired quality of life, as well as suboptimal tolerability of and adherence to cancer treatment. Hormone therapy, performed by increasing estradiol or testosterone levels, is the gold standard for treatment of VMS. However, this approach is generally contraindicated in patients with hormone-sensitive cancers. Nonhormone agents with low to moderate efficacy in controlling VMS are available, but their use may be limited by side effects and tolerability. In this narrative review, the approach to VMS in cancer patients will be discussed. The evidence for various treatment options, including novel agents such as fezolinetant that target the hypothalamic thermoregulatory pathway, will be evaluated. Finally, special considerations in different patient populations based on cancer types (eg, breast, prostate) and age groups (eg, older adults) will be explored.

**Implications for Practice**

Optimal management in cancer survivorship requires timely identification and management of symptoms. Hormone therapy is the gold standard treatment for VMS but is contraindicated in hormone-sensitive cancers. There is growing evidence demonstrating the efficacy of nonhormone options. While generally well tolerated by many, these agents can be associated with side effects that cancer patients may be particularly susceptible to. Encouragingly, emerging drug targets have enabled the development of novel pharmacologic treatments, thereby expanding treatment options for cancer patients with VMS. A comprehensive understanding of these therapeutic agents would enable health care providers to plan effective treatment strategies for cancer survivors experiencing VMS.

## Introduction

An increasing number of people are living with a diagnosis of cancer, due to earlier diagnosis and improved survival of many cancer types.^1^ In many cases, this means that patients are receiving cancer treatments for longer, or living with long-term side effects of cancer treatments. As a result, there is a growing focus on management of side effects of cancer treatment. One common consequence of many cancer treatments is hypogonadism, marked by a reduced level of sex hormones such as testosterone and estradiol. This can occur as a primary treatment goal in the setting of hormone-responsive cancers, as a side effect during gonadotoxic chemotherapy, or as a result of the underlying cancer itself.^2,3^ Hypogonadism is frequently associated with vasomotor symptoms (VMSs), such as hot flashes. Traditionally, VMSs are regarded as hallmark symptoms of menopause in women entering the end of reproductive years.^4^ In the general population, up to 79% of menopausal women and 33% of aging men are affected by VMS.^5,6^ In menopausal women, VMS have a median duration of 7 years, or even longer in those with early onset of VMS, and/or African American race.^7^

The management of VMS is an area of unmet needs in cancer survivorship, and the magnitude of the symptom burden is not to be underestimated.^8^ Globally, breast and prostate cancers, both of which are frequently hormone-sensitive, are amongst the most common cancers. Collectively, they account for 19% of all cancer diagnoses.^9^ The incidence of VMS is particularly high in breast cancer patients on endocrine therapy, including ovarian suppression or ablation, tamoxifen, and aromatase inhibitors (AIs), with a rate of up to 93% of patients depending on treatment type.^10-12^ Anticancer treatment interruption and discontinuation rates for breast cancer patients have been reported to be greater than 20%.^13,14^ The key contributing factor to such poor adherence has been consistently cited as treatment side effects such as VMS.^15,16^ VMSs have also been widely reported in prostate cancer patients on androgen deprivation therapy (ADT).^17^ Unfortunately, the majority of cancer patients with VMS do not receive timely and effective intervention.^8^ Furthermore, hormone therapy (HT), the gold standard treatment for VMS, is often contraindicated in patients with hormone-sensitive cancers.^4^ Prompt recognition and appropriate management of VMS in such patients are integral components of holistic and effective cancer care.

We recognize that hormone-based therapies are used in many different settings in management of cancer and cancer survivorship. For the purpose of this review, terminology will be standardized according to Table 1.

**Table 1.** Terminology and description of hormone-related treatments used in the present review.

Terminology	Description
Hormone therapy	Drugs which aim to increase levels of estradiol or testosterone, including: • Estrogen or combined estrogen and progestogen therapy in women for management of symptoms of menopause, also known as menopausal hormone therapy. • Testosterone therapy in men with hypogonadism.
Endocrine therapy	Drugs which alter the levels of, or block the effects of hormones in the treatment of hormone-sensitive cancers, including: • Ovarian suppression, tamoxifen, and aromatase inhibitors in breast cancer treatment. • Androgen deprivation therapy in prostate cancer treatment.

## Pathophysiology of VMS in cancer patients

Following an abrupt decline in estradiol level in endocrine therapy-treated cancer patients and postmenopausal women, the development of VMS is largely orchestrated by changes in thermoregulatory neurons and the downstream responses.^18^ In prostate cancer patients treated with ADT, suppression of luteinizing hormone (LH) causes decline in testosterone to castrate levels.^19^ As 85% of circulating estradiol in men is derived from peripheral aromatization of testosterone, a decline in testosterone level corresponds to a fall in estradiol.^20^ This process results in a hormonal milieu similar to that in postmenopausal women or women undergoing endocrine therapy. The subsequent steps leading to VMS, however, are not well-established.^21,22^

In healthy individuals, core body temperature range is regulated by physiological and behavioral mechanisms, aimed to achieve a target that would enable smooth performance of body functions.^23^ In individuals with VMS, such homeostasis is altered. This leads to marked narrowing of the thermoneutral zone (defined as the range of temperatures between core thresholds for thermogenesis and thermal dissipation)^24^ and premature triggering of thermoregulatory response, resulting in significant VMS at temperatures well tolerated by others.^25^

**Figure 1.**
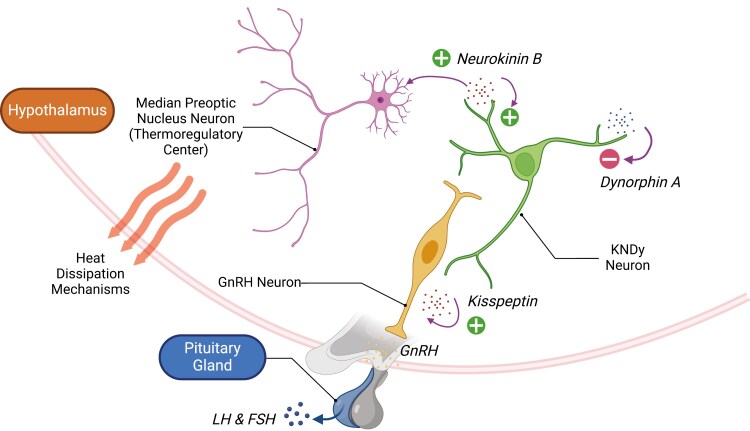
Diagram illustrating the role of the KNDy neuron and its associated signaling pathway in generation of vasomotor symptoms. KNDy neuron is activated by NKB and inhibited by the endogenous opioid peptide dynorphin A. Following menopause, hypertrophy of KNDy neurons leads to increased kisspeptin release, and in turn GnRH secretion and LH pulses. In addition, NKB release is increased, which further stimulates kisspeptin release. NKB also acts on NK3R in the thermoregulatory center to trigger heat dissipation responses, leading to vasomotor symptoms. Abbreviations: FSH, follicle stimulating hormone; GnRH, gonadotropin-releasing hormone; KNDy, Kisspeptin/Neurokinin B/Dynorphin A; LH, luteinizing hormone; NK3R, neurokinin 3 receptor; NKB, neurokinin B.

Following the elucidation of the hypothalamic thermoregulatory pathway, understanding of the pathophysiology of VMS has further expanded. A drop in sex hormone levels leads to an increased expression of kisspeptin by the Kisspeptin/Neurokinin B/Dynorphin A (KNDy) neurons in the arcuate nucleus of hypothalamus.^26,27^ This activates the hypothalamic-pituitary-gonadal axis with increased generation of gonadotropin-releasing hormone (GnRH) pulses in hypothalamic neurons and an increase in secretion of LH. Additionally, the KNDy neurons release neurokinin B (NKB). NKB acts on the neurokinin 3 receptor (NK3R) and activates the thermoregulatory center in the median preoptic nucleus, triggering heat dissipation, vasodilation, and sweating that characterize VMS (Figure 1).^28^

## Clinical assessment of a patient presenting with VMS

A patient with cancer may experience VMS either as result of hypogonadism from endocrine therapy, cytotoxic chemotherapy, surgery or pelvic irradiation; or due to physiological reproductive aging and menopausal transition. The onset of VMS can be accelerated by cancer or cancer treatment. The menopausal transition period is extremely variable in terms of age of onset, duration, and presentation.^29^ Patients’ perception of the symptoms can also be influenced by culture, further increasing the complexity of the assessment.^30^ Thorough history and physical examination are therefore essential when approaching a patient presenting with VMS.

Typically, hot flashes are described as episodes of sudden sensation of extreme heat, originating from the upper chest and face, spreading to the rest of the body and lasting 2-4 minutes. These may be followed by profuse sweating and can also be accompanied by palpitations and anxiety.^4^ As the body temperature drops due to sweating, shivering can ensue. Hot flashes occur more often in the afternoon and night, with varying frequencies ranging from every few days to every hour.^31,32^ Bothersome nocturnal hot flashes and sweating can lead to significant sleep disturbance.^33,34^

While the cause of VMS can be seemingly apparent in a patient receiving cancer treatment with the foreseeable side effects, it is important for clinicians to be mindful of the differential diagnoses that may exist (Table 2).^35^ Fortunately, many of these conditions can be excluded based on history and clinical findings. Among the medical conditions that can cause flashing, discriminating clinical features include onset, duration, associated symptoms, and triggers. Of note, hot flashes that occur with menopause or hypogonadism in cancer patients can present as “wet” flashes (ie, associated with sweating), while flashings mediated by vasoactive substances occur as a result of smooth muscle dilatation and are typically “dry” flashes (ie, not associated with sweating).^36^

**Table 2.** Causes of flashing and sweating disorders.

Endocrinologic conditions	Drugs
Thyrotoxicosis	Vasodilators (eg, nitrates)
Hypoglycemia	Calcium channel blockers
Hypogonadism	Nicotinic acid
Cushing syndrome	Opiates
	Cholinergic drugs
**Neoplasms**	Bromocriptine Cyproterone acetate Cyclosporine Rifampin Sildenafil Tamoxifen Raloxifene Aromatase inhibitors GnRH agonists/antagonists Androgen receptor signaling inhibitors
Medullary thyroid cancer	
Renal cell carcinoma	
Pancreatic neuroendocrine tumor	
Pheochromocytoma	
Carcinoid syndrome	
	**Foods**
**Neurologic conditions**	Caffeine
Anxiety	Spicy food
Migraine	Alcohol
Dysautonomia	Monosodium glutamate
	Sodium nitrite
**Systemic Conditions**	
Systemic mastocytosis	**Cardiovascular conditions**
Polycythemia	Mitral valve disease
Sepsis	

Determination of severity of hot flashes is another key step in clinical assessment. There is no standardized method of defining severity in the clinical setting. Measurement tools, such as the Kupperman Menopausal Index, the Greene Climacteric Scale, and the Menopausal Rating Scale, have been developed to quantify the severity of climacteric symptoms.^37-41^ These tools encompass a broad range of menopausal symptoms and rely largely on patients’ self-reporting. Specific to hot flashes, options include the 7-day Daily Hot Flash Diary that uses a 4-category severity scale (mild, moderate, severe, and very severe) and the Hot Flash Related Daily Interference Scale, both of which have been used in the research of VMS in patients with cancer.^42-44^ A different standard is used for drug approval in the pharmaceutical industry—the Food and Drug Administration (FDA) defines severity of VMS as mild (sensation of heat without sweating), moderate (sensation of heat with sweating, able to continue activity), and severe (sensation of heat with sweating, causing cessation of activity).^45^ This stratification of severity is relatively simple and probably the most practical in the real-world setting.

## Decision-making in management of VMS in cancer patients

Comprehensive guidelines have been developed to guide the management of menopausal symptoms such as VMS.^4,46,47^ These generally recommend HT as first-line therapy due to superior efficacy and tolerability as compared with nonhormone options. However, decision-making may be more complex in cancer patients. In many cases, HT is avoided due to concern for recurrence or growth of hormone-sensitive cancers or risk of venous thromboembolism.^48^ In the subsequent sections, efficacy and safety of hormone- and nonhormone-based management options in cancer patients will be discussed.

## Nonpharmacological management

Lifestyle interventions, such as behavioral adaptations, can be easy to execute and may be considered in those with mild symptoms. These include turning down the thermostat, keeping surroundings cool, using fans or cooling devices, wearing clothing in layers to enable easy adjustment as required, and avoidance of triggers such as spicy food and alcohol.^49,50^ The use of these behavioral strategies is likely widespread, but due to a lack of robust evidence supporting their use, they are not currently recommended as standard management of VMS.^46^

Many observational and interventional studies have explored nonpharmacological strategies for managing VMS. While some studies reported positive outcomes, interpretation and application of the results can be challenging due to heterogeneity in study design, intervention types, patient populations, and outcome measures. Consequently, there is a lack of robust data supporting the dose-response relationship.^46^ Additionally, the short study durations often hinder our understanding of the sustainability of these effects. A summary of the existing evidence and guideline recommendations on a variety of nonpharmacological interventions is listed in Table 3. As a start, promotion of healthy lifestyle forms part of cancer survivorship care and can be initiated in most health care institutions. As menopause transition is a risk factor for cardiovascular disease,^111^ optimization of metabolic health should be recommended to patients as part of holistic cancer management. The efficacy of weight loss and exercise in reduction of VMS, however, has yet been firmly established, hence these strategies should not be promoted primarily as VMS therapy. Cognitive-behavioral therapy, clinical hypnosis, and stellate ganglion block, though shown to have some beneficial effects, require trained personnel and may not be readily accessible to all patients. The evidence for other interventions such as acupuncture, soy food, and black cohosh, is currently insufficient for them to be universally endorsed by major guidelines.^46^ Given the multiple barriers to incorporating research finding into clinical practice, open discussions should be conducted between the clinicians and patients when making treatment decisions.

**Table 3.** Summary of nonpharmacological interventions in treatment of VMS.

Intervention	Background	Summary of evidence	Guideline recommendations
Weight loss	High BMI,^51-53^ weight gain after cancer diagnosis,^54,55^ increased waist circumference^52^ have been identified as possible risk factors for VMS.	Cohort studies evaluating the effect of weight loss on VMS have yielded inconsistent results.^52,56^ Randomized controlled trials have more consistently demonstrated that weight loss from dietary intervention and physical activity could significantly improve frequency and severity of VMS.^57-60^	Recommended by NAMS^46^ (Limited evidence, Levels II-III) and NCCN^48^
Exercise	Early studies showed that exercise triggered VMS in symptomatic women.^25^ Observational studies showed women with regular physical activity were less likely to develop severe VMS^61^	A cochrane review in 2014 and a pooled analysis in 2015 found insufficient evidence to demonstrate the efficacy of exercise in treating VMS.^62,63^ Since then, some studies have shown that yoga, resistance training, training program, and stretching could improve VMS,^64-67^ but others demonstrated no significant benefit.^68^	Not recommended by NAMS^46^ for VMS (insufficient/poor evidence, Level II) Recommended by NCCN^48^
Smoking cessation	In observational studies, former and current smoking have been found to increase risk of VMS in menopausal women^69-71^	Longitudinal data demonstrated that early smoking cessation (before age 40)^71^ and longer duration of smoking cessation (more than 5 years) were associated with lower risk of VMS,^71,72^ but there is a lack of interventional studies to evaluate such effect.	Recommended by ES^4^ to address smoking-cessation during menopause transition
Cognitive-behavioral therapy (CBT)	CBT combines cognitive and behavioral strategies to manage a number of disorders.^73^ A cognitive model has been described to explain symptom perception and behavioral response to VMS.^74^	Evidence from randomized controlled trials supports of the use of CBT in treating VMS, including in breast cancer survivors.^75^ A variety of delivery methods have proven to be effective, including self-guided therapy, remote therapy (via telephone and internet), and group therapy, administered by clinical psychologists or specialist nurses.^76-78^	Recommended by NAMS^46^ (Level I), NCCN,^48^ ESO/ESMO^79^
Clinical hypnosis	Clinical hypnosis is a form of mind-body therapy that makes use of guided technique to achieve a relaxed state. It has been explored in the management of various cancer related symptoms.^80,81^	Randomized controlled studies evaluating clinical hypnosis in postmenopausal women and breast cancer survivors have demonstrated significant reduction of hot flash frequency and score.^82,83^	Recommended by NAMS^46^ (Level 1), NCCN^48^
Stellate ganglion block (SGB)	SGB involves sympathetic block by injecting anesthetic agent at C6 or C7 vertebral level. It has been used to treat a variety of conditions including pain syndrome.^84^ Complications are rare and include injury to nerves, blood vessels, esophagus, trachea, lungs and allergic reactions, etc.^84,85^	Prospective studies evaluating SGB on VMS in postmenopausal women and breast cancer survivors have yielded positive results, but the duration of response to each administration of SGB and timing of repeat block remain unclear.^86,87^	Recommended by NAMS^46^ (Levels II-III)—careful evaluation suggested, given its risks and adverse events
Acupuncture	Acupuncture is a form of complementary therapy that involves applying fine needles or pressure to acupoints of skin.^88^ It has been used to manage a number of cancer related symptoms.^89^	Prospective studies have evaluated the efficacy of acupuncture on VMS in both postmenopausal women and cancer survivors.^90,91^ Acupuncture has been shown to reduce VMS in some but not all postmenopausal women.^92,93^ Factors associated with response to acupuncture have not been consistently established.^94^ When compared with sham acupuncture, the superiority of true acupuncture has not been consistently proven.^95-97^	Not recommended by NAMS^46^ (Level I for traditional acupuncture, Level II for electroacupuncture) Recommended by NCCN^48^ and ESO/ESMO (Level I/B)^79^
Soy foods	Isoflavones from soy food products are phytoestrogens which bind to ERs, with greater affinity for ERα than ERβ. They have estrogen-agonist and estrogen-antagonist properties.^98^ Questions have previously been raised regarding phytoestrogen consumption and breast cancer risk.^99^	Soy consumption has been demonstrated to have either a benefit^100^ or no significant effect on VMS.^101,102^ Considerations in cancer patients: In breast cancer patients, overexpression of genes involved in cell proliferation was seen in those taking soy supplementation.^103^ However, recent studies including the Global Cancer Update Programme suggested that soy intake was associated with a reduced breast-cancer specific mortality and recurrence.^104,105^	Not recommended by NAMS^46^ (Level II)
Black cohosh	Black cohosh, or *Actaea racemosa L*, is a herbal supplement belonging to the buttercup family.^106^ The mechanism of action of black cohosh appears to be mediated via the opioid receptors, as opposed to ER which was previously thought to be a candidate.^106^	A cochrane review in 2012 concluded that there was insufficient evidence for black cohosh in treating VMS.^107^ Following cases of hepatotoxicity being reported in black cohosh users, cautionary statement is required for black cohosh products.^108^ Considerations in cancer patients: No known association between black cohosh and increased breast cancer risk has been demonstrated.^109,110^	Not recommended by NAMS^46^ (Level I)

Abbreviations: BMI, body mass index; CBT, cognitive-behavioral therapy; ER, estrogen receptor; ES, endocrine society; ESO/ESMO, European School of Oncology/European Society of Medical Oncology; NAMS, North American Menopause Society; NCCN, National Comprehensive Cancer Network; SGB, stellate ganglion block; VMS, vasomotor symptom.

## Pharmacological management

### Hormone therapy

Currently, HT is the gold standard for management of VMS in postmenopausal women, provided its use is not contraindicated (eg, in patients with certain cancer types).^4^ When compared with placebo, oral HT has been shown to lead to 75% reduction in the frequency of hot flashes.^112^ In women with previous hysterectomy, an estrogen-only preparation is the HT of choice. In women with an intact uterus, a progestogen is prescribed with estrogen to reduce the risk of endometrial hyperplasia.^47^ The benefits of HT extend beyond its efficacy in improving VMS, particularly in younger women with surgical menopause. For instance, it has been shown to effectively reduce genitourinary symptoms and prevent fractures.^4^

The concern with using HT arose following the early findings of the Women’s Health Initiative (WHI), which evaluated the benefits and risks of conjugated equine estrogens combined with medroxyprogesterone acetate (MPA), compared with placebo in healthy postmenopausal women.^113^ Patients in the intervention arm were found to have increased risks of coronary heart disease, breast cancer, stroke, and venous thromboembolic disease, calling for an early halt of the trial.^113^ Subsequent post-hoc analyses and extension studies of the WHI, together with large-scale observation studies, enabled better understanding of the risk-vs-benefit profile of different HT types and patient populations. Regarding breast cancer risk, a meta-analysis of 58 studies demonstrated increased breast cancer risk with all types of HT, with the exception of vaginal estrogen. The risk was greater with the use of combined estrogen-progestogen than with estrogen-only preparations and with longer duration of HT.^114^ Of note, risk profile of HT is determined not only by the type and duration of hormone used but also by the route of administration. For instance, transdermal estrogen bypasses the first pass metabolism in the liver, and evidence suggests that it does not significantly increase the risk of venous thromboembolism.^115,116^

Tibolone is another hormone-based agent that is used to treat VMS. It is currently not approved by the FDA and hence not available in the United States.^117^ It is a synthetic steroid that has estrogenic, progestogenic, and androgenic effects.^118^ Because of its progestogenic effect, additional progestogen is not required.^118^ Tibolone has been shown to be more effective than placebo but less effective than HT in reducing VMS.^119^ It carries several risks, such as an increased risk of incident breast cancer in women with history of breast cancer, but not in women without prior history of breast cancer.^119,120^ There is also evidence suggesting that it increases the risk of stroke.^119^ It is currently not recommended as first-line treatment of VMS.^4^

Taking the findings of the abovementioned studies into account, systemic HT is generally not recommended to treat VMS in patients with hormone-sensitive cancers or estrogen receptor positive tumors (such as certain breast, endometrial, and ovarian cancers).^4,47^ Low-dose vaginal estradiol is now a feasible consideration as second-line therapy for urogenital symptoms in patients with history of breast cancer, but it is not an effective treatment for VMS.^121^ In patients with active prostate cancer, testosterone therapy should be avoided. Certain guidelines, however, have now given some provision for exploration of testosterone therapy as a treatment for hypogonadism in patients with treated low-risk prostate cancer, although the limitation lies in the lack of long-term safety data.^19,122^ While studies evaluating the use of estrogen-containing HT in men with VMS demonstrated significant improvement in symptoms, gynecomastia was common side effect.^17^ Progestogen-based treatments such as megestrol acetate and MPA have also been shown to effectively alleviate VMS in both breast cancer and prostate cancer patients.^123-125^ A 400 mg dose of intramuscular MPA, for example, has been demonstrated to provide sustained improvement in VMS in a cohort that included breast cancer survivors during the 6 weeks of observation.^124^ Prostate cancer patients receiving oral MPA of 20 mg daily achieved greater reduction of VMS than those receiving venlafaxine.^125^ Logically, any form of HT should only be prescribed after thorough discussion with the patient, and full disclosure of risks versus benefits. More detailed information pertaining to the safety of HT in each cancer type, and guideline recommendations can be found in Table 4.

**Table 4.** Considerations and guideline recommendations on use of HT in VMS management based on cancer type.

Cancer type	Evidence and considerations	Relevant guideline recommendations
Breast cancer	Safety of HT in disease-free patients with previously treated breast cancer: Increased risk of new breast cancer event: • HABITS (*N* = 442): Duration of HT 2 years. (E or E + P) vs non-HT. At median 4 years follow-up, HR = 2.4, 95% CI, 1.3-4.2^126^ Of note: HT arm had increased risk of local recurrences or contralateral breast cancer, but not distal metastasis. • LIBERATE (*N* = 3098): Tibolone vs placebo. At median 3.1 years follow-up, HR = 1.40, 95% CI, 1.14-1.70^120^ No significantly increased risk of new breast cancer event: • Stockholm trial (*N* = 378): Mean duration of HT 2.6 years. (E or E + P (MPA)) vs vaginal estrogen. At median 10.8 years follow-up, HR = 1.3; 95% CI, 0.9-1.9^127^ Meta-analyses and systemic reviews of interventional and observational studies evaluating the effect of HT on risk of recurrence of breast cancer or new cancer events have led to inconsistent results.^128-130^	Systemic HT not recommended: • ES 2015^4^ • ACO/ASCO 2016^131^ • NAMS 2022^47^ *(If VMS severe and unresponsive to nonhormone options, HT may be considered following discussion with oncologist and assessment of risk and benefits)* • NCCN 2022^48^
Endometrial cancer	A Cochrane Review of 7 RCTs and 2190 participants concluded that there a lack of high-quality evidence on the safety of HT in patients with previously treated endometrial cancer.^132^	Systemic HT not recommended: • ES 2015^4^ • NAMS 2022^47^ *(Not recommended in high-grade, advanced-stage endometrial cancers or in endometrial stromal sarcomas or leiomyosarcomas. HT may be used to treat bothersome VMS in women with low-grade, stage I endometrial cancer after hysterectomy, if no improvement with nonhormone therapies, following consultation with oncologist.)* • NCCN 2022^48^ *(Relatively contraindicated in survivors high-risk endometrial cancer)*
Ovarian cancer	Systematic reviews and meta-analyses reviewing use of HT in ovarian cancer survivors did not report reduced survival in those who received HT.^133,134^ There is currently a lack of high-quality data detailing the safety of HT in specific subtypes of ovarian cancers.	Systemic HT not recommended: • ES 2015^4^ • NAMS 2022^47^ *(Not recommended in hormone-dependent cancers including granulosa-cell tumour and serous carcinoma. HT may be considered in symptomatic women with tumours of low-malignant potential which have been completely resected.)*
Prostate cancer	Testosterone replacement: There is currently a lack of good-quality data evaluating the safety of androgen in patients with history of prostate cancer, but an RCT is underway to assess this.^135^ Other types of HT: • Data from RCT reported effectiveness of medroxyprogesterone acetate and cyproterone acetate for treatment of VMS in prostate cancer patients on GnRH analogue.^125^ • While megestrol acetate is effective in reducing VMS,^136^ safety appears variable—most studies report good tolerability and safety, but there are cases reporting rising PSA while on megestrol acetate^123,137^	Testosterone not recommended: • ES 2018^138^ • NCCN 2022^48^ Other comments: • EAU 2024^19,139^ - Androgens contraindicated in men with active prostate cancer. Possible use of testosterone therapy in symptomatic hypogonadal men with previously treated prostate cancer which are EAU low-risk for recurrence, after full discussion with patient. *Note: this comment pertains to hypogonadism and is not specific to VMS Regarding use of other types of HT and agents • NCCN 2022^48^ [*HT (medroxyprogesterone, cyproterone acetate, estrogen) in appropriate candidates with referral to appropriate specialists.]* • CUA 2022^140^ *(Agents which may be considered: medroxyprogesterone acetate, megestrol acetate, cyproterone acetate, gabapentin, venlafaxine.)*
	*Collectively these recommendations highlight the concerns with using testosterone in patients with history of prostate cancer, as well as lack of long-term safety data. While HT may be considered for low-risk patients with successfully treated prostate cancer and undetectable PSA level, a watch period to ensure absence of evidence of disease is required before HT commencement.*	

Abbreviations: ACO/ASCO, American Cancer Society/American Society of Clinical Oncology; CEE, Conjugated Equine Estrogen; CUA, Canadian Urological Association; E, Estrogen; E + P, Estrogen and Progestogen; EAU, European Association of Urology; ES, Endocrine Society; HABITS, Hormonal Replacement After Breast Cancer—Is it Safe?; HR, Hazard Ratio; LIBERATE, Livial Intervention following Breast cancer Efficacy, Recurrence, and Tolerability Endpoints; MPA, Medroxyprogesterone Acetate; NAMS, North American Menopause Society; NCCN, National Comprehensive Cancer Network; PSA, Prostate-Specific Antigen; RCT, Randomized Controlled Trial; WHI, Women’s Health Initiative.

For most patients with history of a cancer that is not hormone-sensitive, on the other hand, HT can be a viable option. It should be actively considered in younger women and childhood cancer survivors to prevent bone loss from estrogen deficiency.^141^ Nonetheless, by virtue of the systemic effects of cancer and sequelae of cancer treatments, cancer patients may be more susceptible to adverse events of HT. For example, cancer patients are at increased risk of developing venous thromboembolism and cardiovascular events.^142-144^ Women with previous chest radiation for Hodgkin lymphoma have increased risk of breast cancer.^145^ Furthermore, increased breast cancer risk has been well-described in a number of populations, including those with known mutation in BRCA 1 or BRCA 2 gene and those with certain familial cancer syndromes such as Li-Fraumeni syndrome.^146,147^ Reassuringly, some of these concerns have been addressed by prospective studies. Observational evidence suggests that HT use does not further increase risk of breast cancer in BRCA 1 and BRCA 2 mutation carriers without known cancer, who are status-post risk-reducing bilateral salpingo-oophorectomy.^148,149^

The current consensus states that in the majority of low-risk VMS patients from the general population, below the age of 60 or within 10 years of menopause onset, the benefits of HT will outweigh the risks.^4^ Given the complexity of the management considerations in cancer patients however, HT should be offered only after careful assessment of risks versus benefits. The Endocrine Society suggests exercising caution when considering HT in patients with intermediate breast cancer risk, and to avoid HT in those with high risk for breast cancer, as stratified by the National Cancer Institute Breast Cancer Risk Assessment Tool.^4,150^ If a decision for HT is made, it should be prescribed based on recommendations from societal guidelines. The dose of HT used should be the lowest needed to achieve therapeutic response.^4,47^

### Nonhormone therapy

There is growing evidence supporting the utilization of nonhormone treatments in cancer patients with VMS. Pharmacological agents that have been evaluated include antidepressants, GABA analogues, clonidine, oxybutynin, etc. The onset of action of these drugs is rapid, usually within 1-2 weeks.^46^ When examining the efficacy of these agents, it is important to note the significance of placebo effect in trials. Typically, placebo alone can achieve ~20%-60% improvement in outcome measures in VMS studies.^46^ Interestingly, a lower placebo response is seen in studies with breast cancer patients.^151^ As many of these drugs target neurotransmission pathways, older adults can be particularly susceptible to adverse effects. Currently, selective serotonin reuptake inhibitors (SSRIs), serotonin norepinephrine reuptake inhibitors (SNRIs), gabapentin, and fezolinetant are the agents preferentially recommended by NAMS with level I evidence and oxybutynin with level I-II evidence.^46^ Suggested considerations in prescribing these drugs are summarized in Table 5.

**Table 5.** Summary of pharmacological interventions in treatment of VMS.

Medication	Commonly used dose	Considerations in specific patients populations
Hormone therapy	*Note: many options are available, for more detailed description of regimens, refer to ES^4^ and NAMS^47^ guidelines Systemic estrogen therapy—Transdermal estradiol patch (0.025-0.1 mg) once or twice weekly With progestogen therapy in women with intact uterus—Oral micronized progesterone 200 mg once daily cyclically (~12 days for 28-day cycle) or 100 mg once daily continuously	Special patient population (see Table 4 for details) • Cancer patients—HT generally contraindicated in hormone-sensitive cancers • Older adults—avoid starting in women over the age of 60 and more than 10 years from menopause onset Other contraindications include unexplained vaginal bleeding, history of cardiovascular disease, venous thromboembolism, arterial thromboembolic disease, liver impairment, thrombophilic disorders, increased risk of cardiovascular disease, increased risk of breast cancer, etc. (list is not exhaustive, refer to societal guidelines for further details).^4,47^
Antidepressants	Escitalopram (SSRI)—start with 5-10 mg once daily, increase to 20 mg once daily after 4 weeks as needed Citalopram (SSRI)—start with 10 mg once daily, increase to 20 mg once daily after 1 week as needed Paroxetine (SSRI)—start with 7.5 mg (single dose capsule with no titration needed) or 10 mg/day, increase to 25 mg/day as needed, depending on type of preparation Venlafaxine (SNRI)—start with 37.5 mg/day, increase to 75 mg/day as needed	Special patient population • Cancer patients—hyponatremia can be associated with both cancer and SSRI use.^152,153^ Paroxetine has weak anticholinergic effect and may cause/worsen dry mouth and constipation.^154^ Both SSRI and SNRIs can cause nausea soon after initiation, but symptom usually improves.^155^ • Breast cancer patients—avoid paroxetine in patients taking tamoxifen.^156^ • Older adults—higher risk of dose-dependent increase in BP induced by SNRI,^157^ hyponatremia induced by SSRI,^158^ QT interval prolongation induced by citalopram and escitalopram,^159^ risk of symptomatic withdrawal if patient has tendency to omit/stop medication (especially with paroxetine and venlafaxine)^160^ Contraindications: SSRIs and SNRIs contraindicated in patients with history of serotonin syndrome, and current use of monoamine oxidase inhibitors.^46^ Drug interactions: antiemetics (5HT3 antagonists such as ondansetron), monoamine oxidase inhibitors, opioids^161^
GABA analogues	Gabapentin—start with 100-300 mg/day with first dose given at bedtime, gradual increase to 900 mg/day in divided doses as needed. Higher doses may be potentially given, but most trials use dose up to 900 mg/day	Special patient population • Older adults—gabapentin is generally safe, but may cause dose-dependent dizziness or drowsiness.^162^ Use with caution in patients at risk of falls. Consider evening dosing to minimize impact of adverse effects. Start low dose and increase slowly. • Renal impairment—Dose adjustment is needed in patients with renal impairment. Drug interactions: opioids (may enhance CNS depression effect)
Clonidine	0.05 mg twice daily. Discontinue treatment if no improvement after 2-4 weeks	Special patient population • Older adults—clonidine can cause hypotension and bradycardia.^163,164^ Use with caution in patients at risk of falls. Drug interactions—beta blockers (risk of bradycardia), hydroxyzine (may enhance CNS depression effect)^165^
Oxybutynin	Immediate release 2.5-5 mg twice/day or Extended release 5-15 mg/day	Special patient population • Older adults—increased risk of anticholinergic effects such as CNS disturbance (confusion, agitation, cognitive impairment, drowsiness, dizziness), urinary retention, constipation, visual disturbance, tachycardia^166^
Fezolinetant	45 mg/day	Newly approved and limited data available. From SKYLIGHT 1 trial, treatment-emergent adverse reactions included headache and a small number of participants with abnormal ALT or AST.^167^ It is recommended to measure liver enzyme levels at baseline and monthly for the first 3 months, then at months 6 and 9 following initiation of treatment.^168^ Contraindications: avoid in patients with cirrhosis, severe renal impairment, or end stage renal disease. Drug interactions—Fezolinetant is metabolized by CYP1A2 and should be avoided in patients on CYP1A2 inhibitors^169^

Abbreviations: ALT, alanine transaminase; AST, aspartate aminotransferase; CNS, central nervous system; RCT, randomized controlled trial; SNRI, serotonin–norepinephrine reuptake inhibitor; SSRI, selective serotonin reuptake inhibitor.

#### Antidepressants

Selective serotonin reuptake inhibitors (eg, escitalopram, citalopram, and paroxetine) and SNRIs (eg, duloxetine and venlafaxine) are the largest groups of nonhormone therapies studied, both in healthy women and cancer patients with VMS.^170-172^ They can reduce hot flash frequency by up to 58% and hot flash scores by up to 65%.^170,171,173^ Selective serotonin reuptake inhibitors and SNRIs have also been compared with other drugs such as clonidine.^174^ As there is no convincing evidence supporting the superiority of any these agents over others, tolerability and side-effect profile are important determinants of treatment outcome. At present, paroxetine (Brisdelle, 7.5 mg) is the only antidepressant approved by the FDA for the treatment of moderate-to-severe VMS associated with menopause.^175^ Its efficacy has also been demonstrated in patients with breast and gynecological cancers.^172,176^ However, due to its strong inhibition of the cytochrome P450 2D6 (CYP2D6) enzyme, paroxetine should be avoided in patients on tamoxifen treatment. Active metabolites of tamoxifen can be decreased by up to 64% in patients taking paroxetine.^156^ Likewise, fluoxetine, another strong CYP2D6 inhibitor, should also be avoided in tamoxifen-treated patients. In older adults, venlafaxine and paroxetine may not be the initial drug of choice due to risk of withdrawal.^160^ Escitalopram and citalopram may be the preferred drugs in this age group due to their lower side effect profile. Compared with citalopram, there are fewer cardiac side effects with escitalopram (eg, QTc prolongation).^177,178^ Drowsiness and dizziness are common adverse effects of SSRIs and SNRIs and can be potentially managed by adjusting the timing of administration.^179^ Taking the medication at night may not only alleviate symptoms from nocturnal VMS more effectively but also mitigate side effects such as nausea. Caution should be exercised in patients with hyponatremia, which can be associated with both cancer and SSRI use.^152,153^ Other adverse effects of antidepressants include sexual dysfunction and weight gain, which may overlap with symptoms of menopause, and should be considered in discussion on VMS treatment options.^180,181^ Regardless of the drug chosen, the lowest possible dose necessary for symptom relief should be used. Upon decision to stop the therapy, tapering should be done to avoid withdrawal symptoms.^179^

#### GABA analogues

γ-Aminobutyric acid (GABA) analogues such as gabapentin and pregabalin have been studied in the treatment of VMS in patients with and without cancer. Gabapentin, but not pregabalin, is currently recommended by NAMS for treatment of VMS.^46^ At a dose of 900 mg per day, gabapentin can reduce hot-flash frequency by 44% and severity by 46% in breast cancer patients.^182^ This effect appears to be dose dependent, as the 300 mg per day treatment arm achieved a smaller improvement in frequency and severity scores. A trial of pregabalin demonstrated reduction of hot flash score by 65% in the 75 mg twice daily regimen, and by 71% in the 150 mg twice daily regimen. These effects, however, are on a background of 50% placebo response.^183^ In addition to alleviating VMS, gabapentin has also been shown to improve anxiety scores in breast cancer survivors with anxiety.^184^ Considering these findings and its neuropathic pain-alleviating property, gabapentin is a viable option in cancer patients with VMS and pain or anxiety. Weight gain is a potential adverse effect which should be discussed with patients.^185^

#### Clonidine

Clonidine is a centrally acting α2-adrenergic agonist primarily used in treatment of hypertension and attention deficit hyperactivity disorder. The ability of clonidine, both oral and transdermal, to reduce the frequency of hot flashes was demonstrated decades ago.^186-188^ In breast cancer patients with VMS, clonidine can reduce the frequency of hot flashes by 38%.^189^ Dry mouth, lethargy, nausea, and difficulty in sleeping are the most frequently reported side effects.^174,188,190^ Owing to its ability to reduce blood pressure, clonidine may be considered in patients with concomitant hypertension but should be used with caution in patients who are prone to dizziness and falls, such as older adults.

#### Oxybutynin

Oxybutynin, an anticholinergic drug primarily prescribed for overactive bladder, can effectively reduce hot flash scores and frequency when administered at doses of 2.5 and 5 mg twice daily.^191^ However, its anticholinergic properties give rise to side effects such as dry mouth, difficulty urinating and constipation, all of which may be particularly problematic in older adults. Additionally, oxybutynin can increase risk for falls and delirium in this population.^192^ When a dose of 15 mg daily dose was used, the rate of adverse events increased significantly, leading to drug discontinuation in 6.8% of participants in a randomized controlled trial.^193^

#### Fezolinetant

Following the elucidation of the role of KNDy neuron in the development of VMS, neurokinin receptors naturally became potential drug targets for management of VMS. Fezolinetant, a selective NK3 receptor antagonist, demonstrated greater than 61% in reduction of hot-flash frequency in SKYLIGHT I trial.^167^ Fezolinetant is now approved by the FDA for treatment of moderate to severe hot flashes, at a dose of 45 mg once daily.^194^ Encouragingly, a recent meta-analysis revealed that fezolinetant reduced VMS frequency, demonstrating comparable efficacy to HT, and significantly exceeding the reduction seen in other nonhormone agents.^195^ Furthermore, longer studies have shown that the drug appeared to be safe with a low rate of drug discontinuation.^196,197^ Adverse effects of fezolinetant include increased alanine transaminase (ALT) or aspartate transaminase (AST) levels, which is typically mild, transient, with most of the ALT and AST elevations being no greater than 3 times the upper limits of normal.^167,198^ At present, there is no data on the effect and safety of fezolinetant in cancer survivors.

#### Other novel agents

A few other drugs have been developed to block the activation of the NK3 receptor. Elinzanetant, a dual neurokinin 1 and neurokinin 3 receptor antagonist, also appeared to be well tolerated in phase II trials, with several phase III trials being conducted currently.^199^ Q-122 is an oral agent, which can reduce KNDy neuron activation independent of the NK3 receptor pathway, and a recent phase 2 study demonstrated significant reduction in VMS compared with placebo.^200^ Notably, the study was conducted in breast cancer patients on tamoxifen or AI. Collectively, the development of these novel therapeutic agents has brought about great promise to the options that cancer patients with VMS may have in the near future.

## Suggested approach in clinical practice

**Figure 2.**
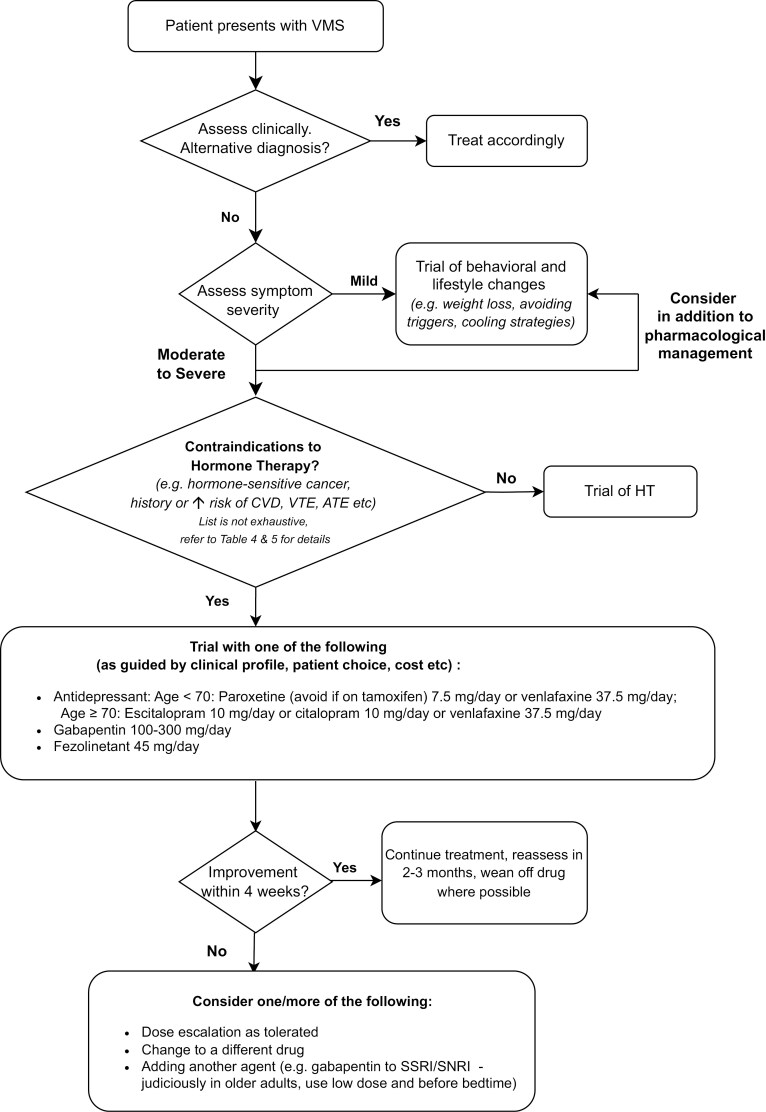
Suggested approach to a cancer patient presenting with VMS. Abbreviations: ATE, arterial thromboembolism; CVD, cardiovascular disease; HT, hormone therapy; SNRI, serotonin norepinephrine reuptake inhibitors; SSRI, selective serotonin reuptake inhibitors; VMS, vasomotor symptoms; VTE, venous thromboembolism.

Based on the current understanding of efficacy and safety of drugs used to treat VMS and the authors’ collective clinical experience, we have proposed the following clinical approach to VMS in cancer patients (Figure 2). As most of the trials on nonhormone therapies have short monitoring intervals of 4 weeks, and follow-up duration of under 6 months, we suggest that patients who are commenced on such treatment be reviewed regularly. If a therapy is deemed to be ineffective at the recommended dose (which is often a low dose), there may be limited value in increasing the dose further. As the overarching aim of management of VMS is to improve the quality of life of cancer survivors, the side effect profile of any pharmacological agent should be weighed against the clinical benefit.

## Conclusion

Given the high prevalence of VMS in cancer patients, it would be valuable for managing clinicians such as oncologists, endocrinologists, and primary care providers to routinely ask about VMS and be familiar with the approach to VMS management. The field is evolving, with richer long-term data now available on the risks and benefits of HT in patients with specific cancer types. Nonhormone therapies have emerged as effective alternatives for many and are now incorporated into major guidelines. Furthermore, the emergence of novel therapies such as fezolinetant has provided additional treatment options, thereby fostering a more promising outlook for cancer survivorship.

## Data Availability

No new data were generated in this research. Data underlying this article are available on request.
